# Supplementary material for: In-air fast response and high speed jumping and rolling of a light-driven hydrogel actuator
Source: Nat Commun. 2020 Aug 10;11:3988. doi: 10.1038/s41467-020-17775-4 (PMC7417580; doi:10.1038/s41467-020-17775-4)
Supplement: Supplementary file 1 — Supplementary Information [file 41467_2020_17775_MOESM1_ESM.pdf]

Supplementary Information

In-air fast response and high speed jumping and rolling of a light-driven hydrogel actuator

Mingtong Li[1,2,†]*, Xin Wang*[1,†]*, Bin Dong*[1,*] *and Metin Sitti*[2,*]

[1] Institute of Functional Nano & Soft Materials (FUNSOM), Jiangsu Key Laboratory for Carbon-Based Functional Materials & Devices, Soochow University, Suzhou, Jiangsu 215123, P. R. China

[2] Physical Intelligence Department, Max Planck Institute for Intelligent Systems, 70569 Stuttgart, Germany

[†] These authors contributed equally to this work.

* Correspondence to: bdong@suda.edu.cn; sitti@is.mpg.de

Supplementary Table 1. Comparison of different types of hydrogel actuator in terms of movement speed and response time.

Types	Maximum movement speed	Response time	Ref.
PAANa-IONP hydrogel actuator in the current study	1.6 m/s	0.8 s	
Spiropyran based hydrogel actuator	15.2 μm/min	5 min	35
PEGDA hydrogel actuator	26.07 cm/s	4.6 s	49
Graphene-elastin composite hydrogel actuator	1 mm/s	10 s	51
PNIPAM hydrogel actuator	4.4 μm/s	1 s	34
PNIPAM-WS_2 hydrogel actuator	1 mm/s	6 s	38
Poly(NIPAM-DMAPMA) bilayer hydrogel actuator	9.61 mm/s	420 s	42

Supplementary Figures

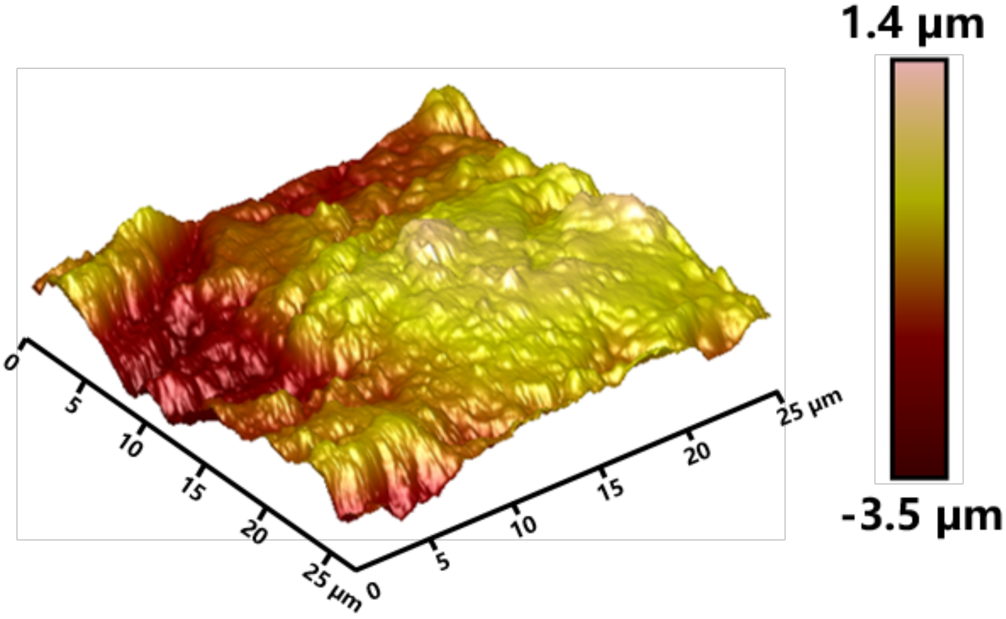

Supplementary Figure 1. The AFM image showing the surface morphology of the as-synthesized crosslinked PAANa and IONP composite.

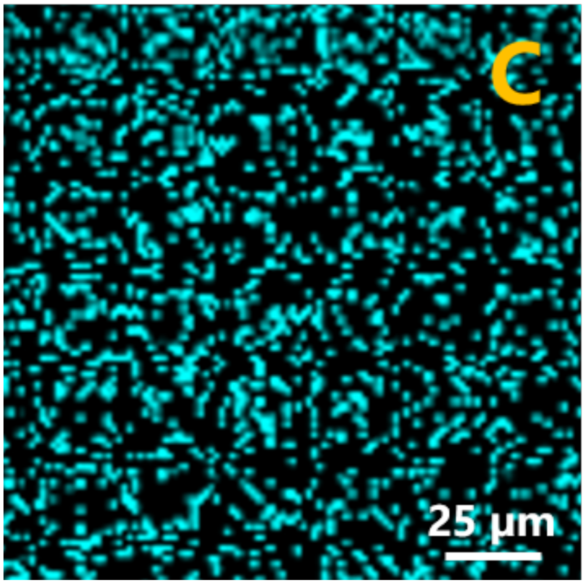

Supplementary Figure 2. The corresponding EDX analysis for carbon element of the SEM image shown in Fig. 1e.

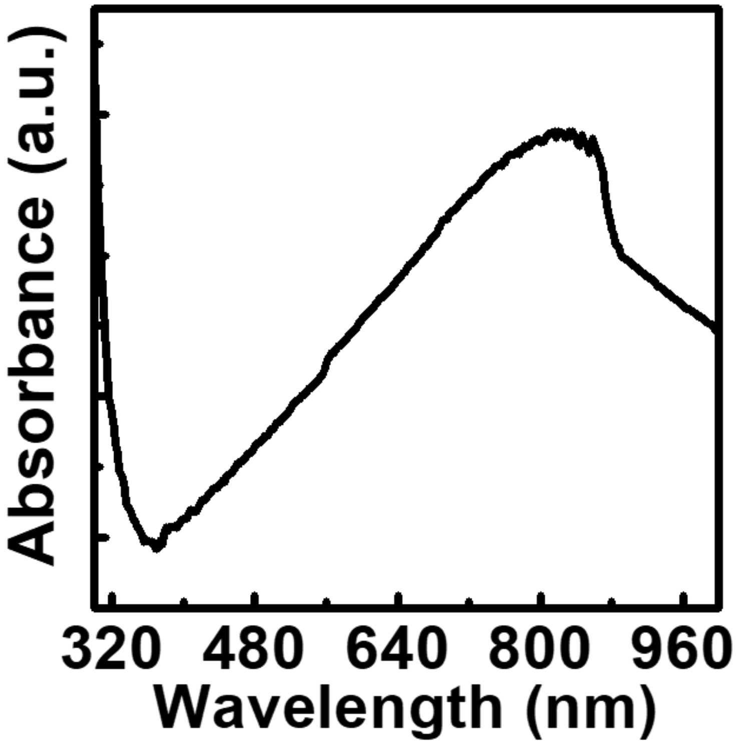

Supplementary Figure 3. The UV-Vis-NIR spectrum of the as-synthesized PAANa-IONP composite hydrogel.

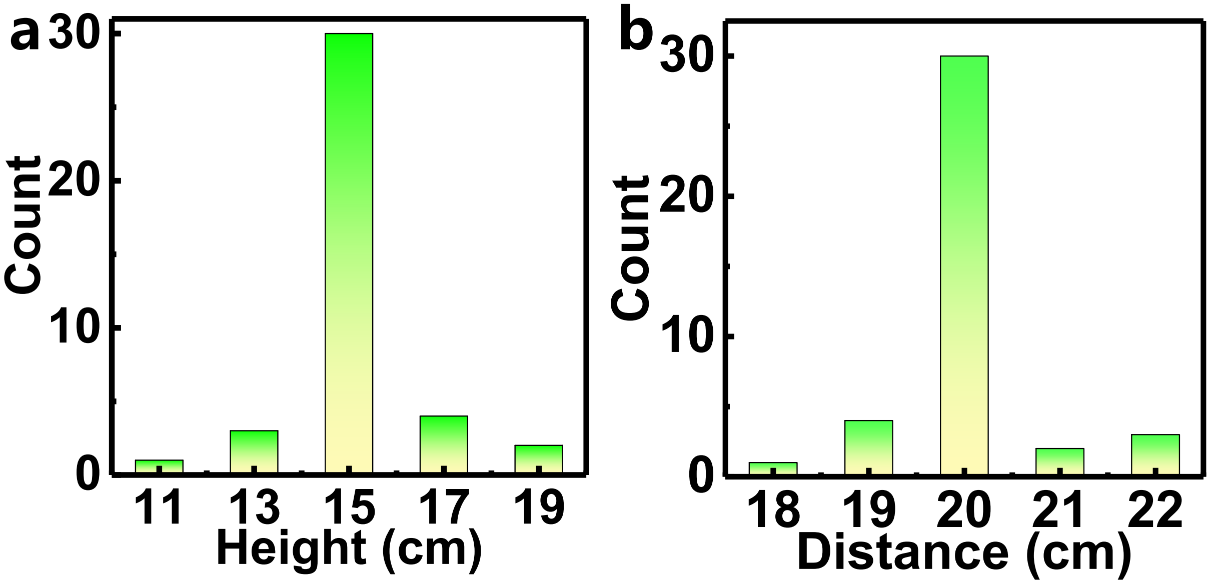

Supplementary Figure 4. (a) The jumping height and (b) rolling distance of the hydrogel actuator upon 2.34 W and 0.67 W light actuation, respectively.

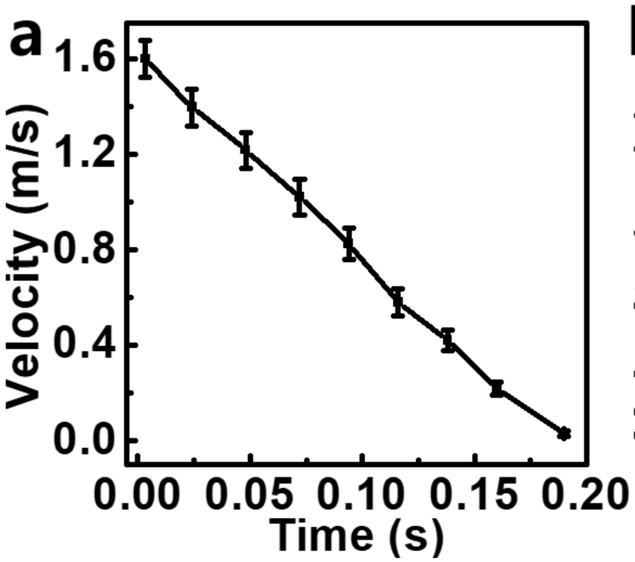

Supplementary Figure 5. The velocity change during (a) jumping and (b) rolling of the hydrogel actuator after 2.34 W and 0.67 W light actuation, respectively. Note that, for (a), the jumping actuator is in the air when the velocity decreases to zero. Error bars denote the standard deviation.

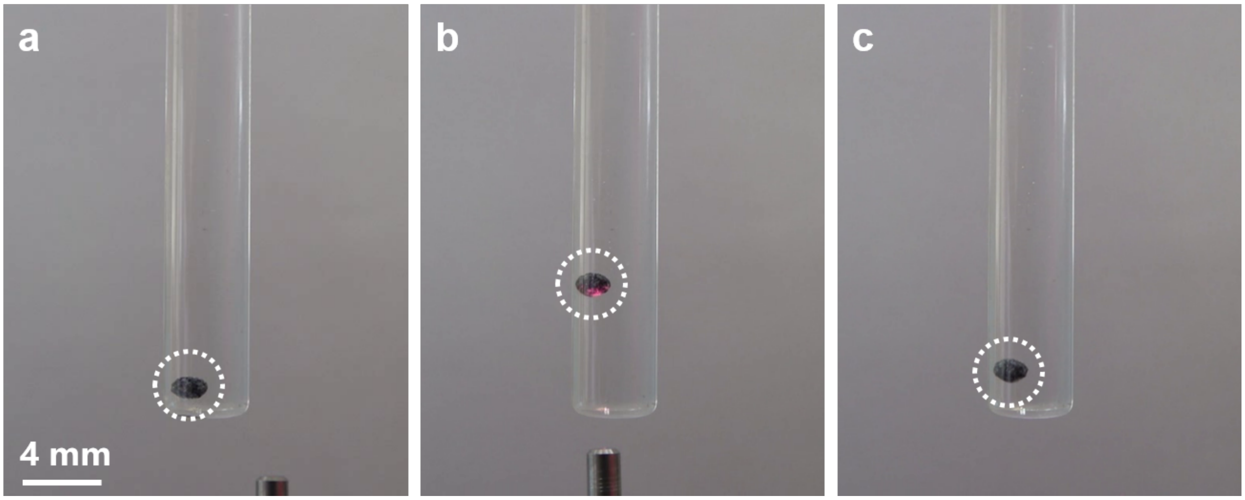

Supplementary Figure 6. (a) The hydrogel actuator at the bottom of the solution before the light irradiation. (b) The surfacing process of the hydrogel actuator after 2.34 W light irradiation. (c) It falls back to the bottom of the solution once the light irradiation is withdrawn. These pictures are obtained from the Supplementary Video 4.

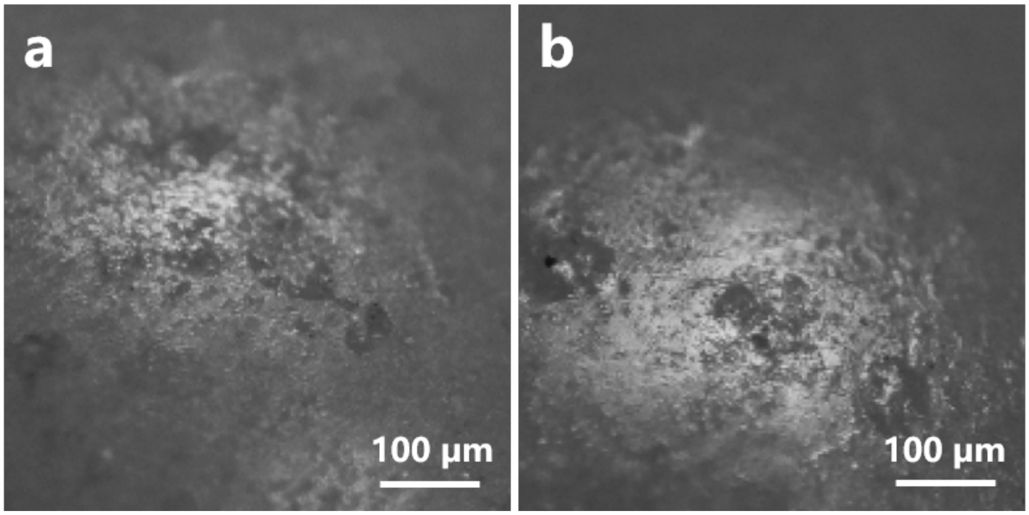

Supplementary Figure 7. Optical microscopic images showing the surface morphology of the hydrogel actuator (a) before and (b) after jumping (under 2.34 W NIR irradiation).

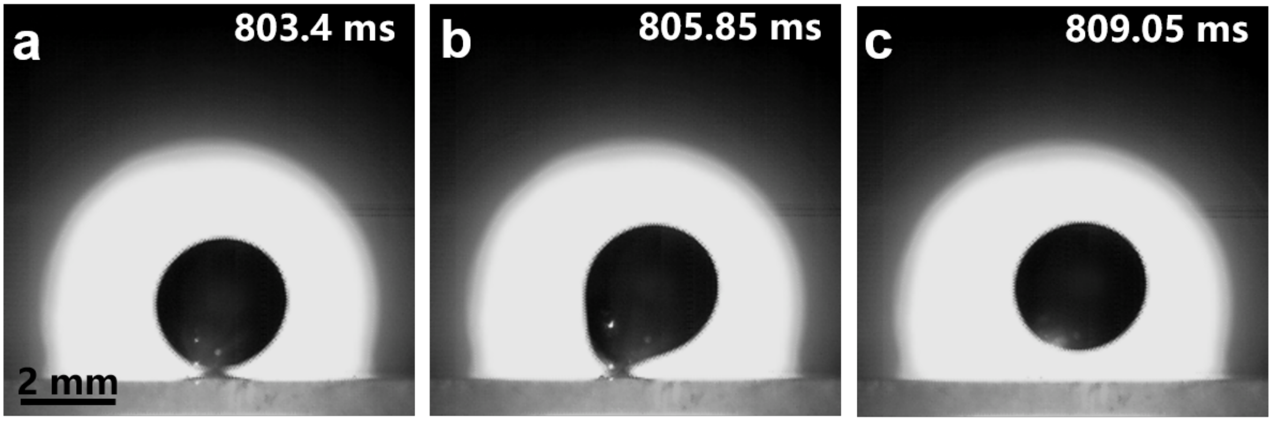

Supplementary Figure 8. (a-c) A series of images recorded by high speed camera showing the take-off process of the hydrogel actuator without the PBR surface coating under 2.34 W laser irradiation. These images are captured from Supplementary Video 6.

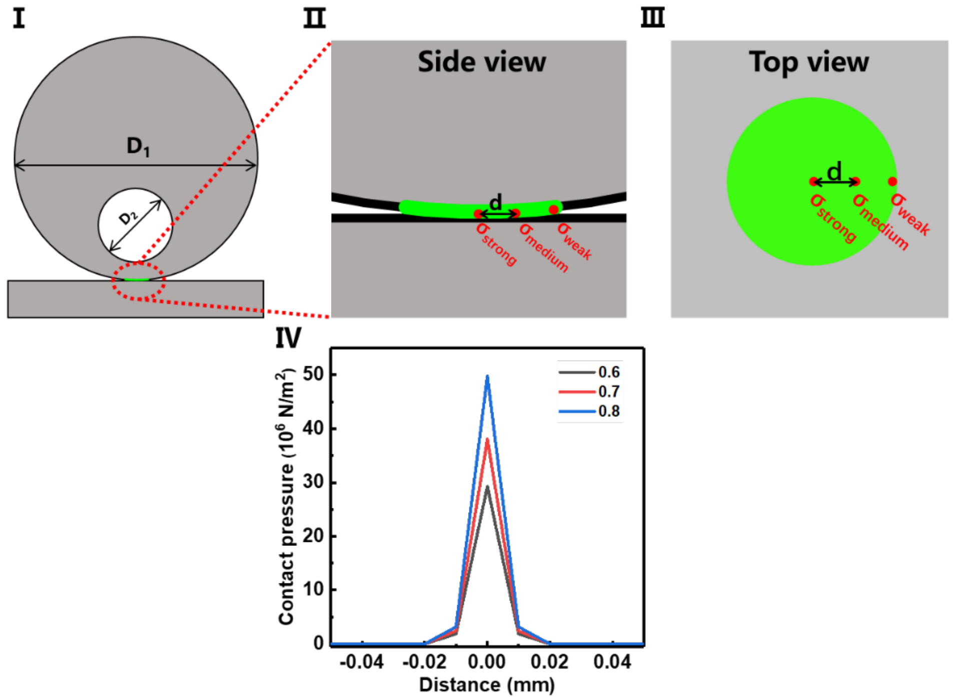

Supplementary Figure 9. (I) The illustration showing the numerical simulation and (II) side view and (III) top view of the contact area. (IV) The contact pressure (σ) versus the distance from the center of contact (d) between the hydrogel actuator and the substrate for different bubble cavity diameters (0.6 mm, 0.7 mm and 0.8 mm) obtained by the simulation method. Note that the distance to the left is defined as negative.

According to equation 2: $F_{jumping} = \sigma S$, the force depends on S, i.e. the contact area, which is illustrated in (I-III) as the green colored area. d is the distance from the center of contact to a certain position inside the contact area (II-III). And the contact pressure (σ) inside the contact area is different at different location, i.e. the contact pressure is the highest at the center (σ_{strong}), medium in the middle (σ_{medium}) and the smallest at the edge (σ_{weak}). (IV) shows the typical contact pressure distribution inside the contact area as a function of d. Therefore, the total force can thus be obtained by the integral of the contact pressure over the entire contact area (III).

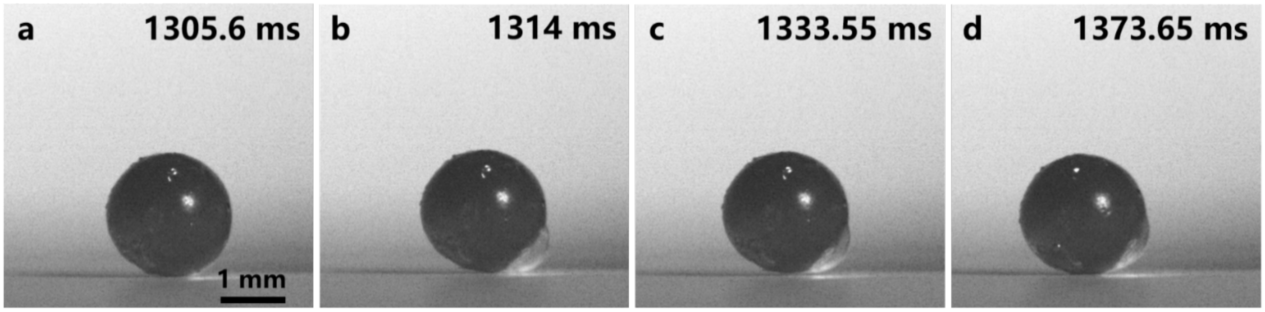

Supplementary Figure 10. (a-d) Snapshots of a high-speed camera video recording showing the actuator starts rolling when irradiating its side part with 0.67 W light. These pictures are obtained from Supplementary Video 7.

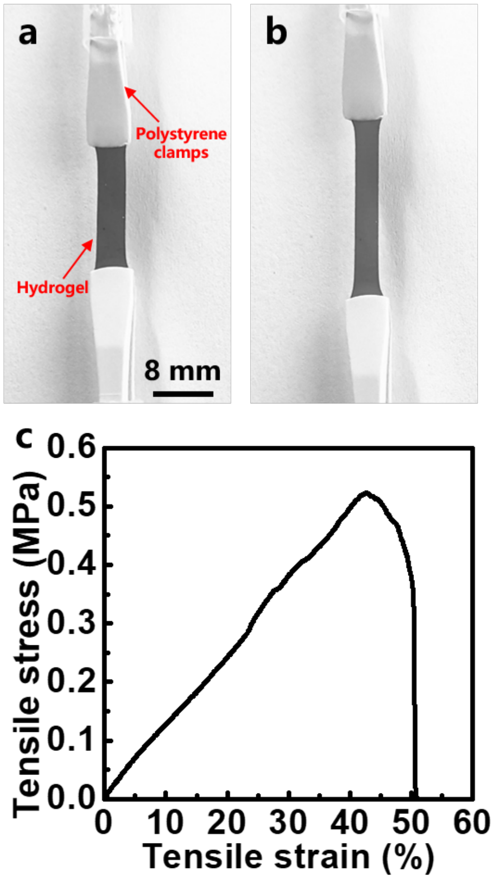

Supplementary Figure 11. A stripe-shaped hydrogel containing 2 wt% IONP (a) before and (b) after stretching (42 % elongation). (c) Tensile stress-strain curve of the hydrogel containing 2 wt% IONP.

We have synthesized the hydrogel comprising the actuator (containing 2 wt% IONP) in the shape of a stripe and studied its mechanical property by utilizing the Instron tensile tester. As can be seen from (c), the fracture of the hydrogel containing 2 wt% IONP occurs when the strain is higher than 42 %. According to the numerical simulation (the same simulation shown in Fig. 2o), the maximum strain of the hydrogel actuator during bubble formation is estimated to be around 20 %, which is much lower than the fracture strain of the hydrogel. Therefore, the hydrogel does not fracture during the bubble formation process.

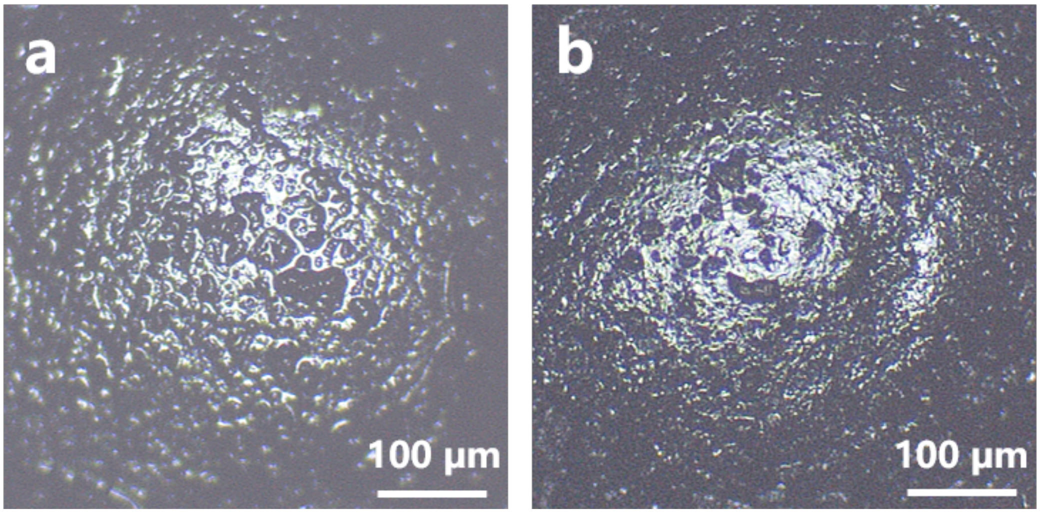

Supplementary Figure 12. Optical microscope images showing the surface morphology of the hydrogel inside the hydrogel actuator after peeling of the surface PBR coating: (a) before and (b) after NIR actuation (2.34 W).

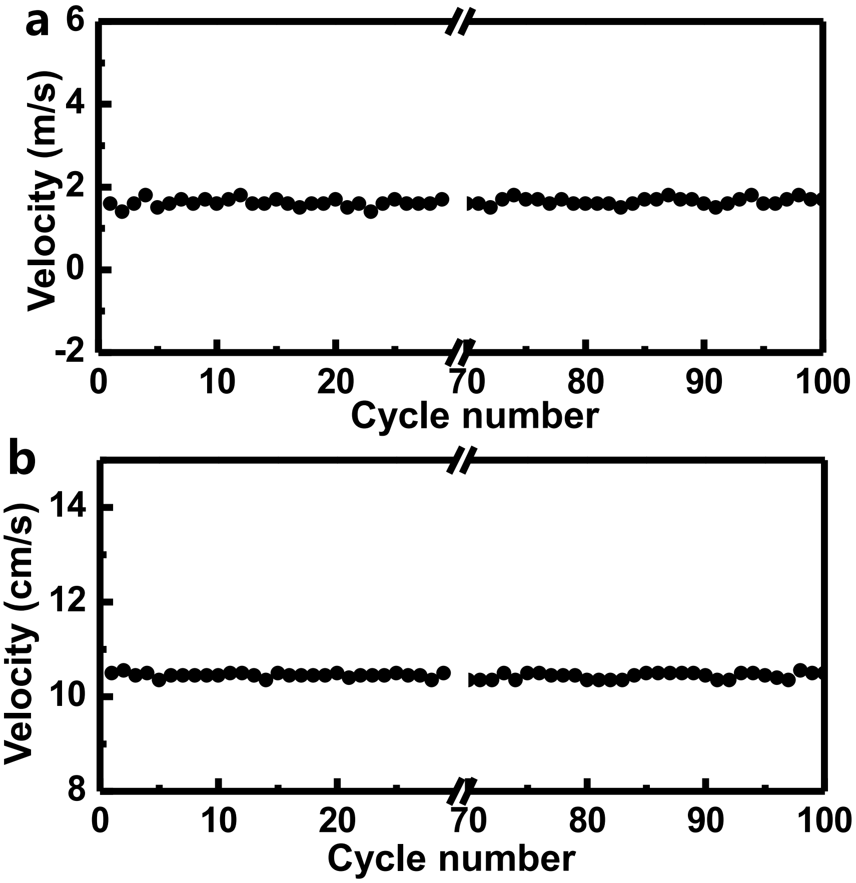

Supplementary Figure 13. The repeatability of (a) jumping and (b) rolling behavior of the hydrogel actuator upon 2.34 W and 0.67 W repetitive light irradiation, respectively.

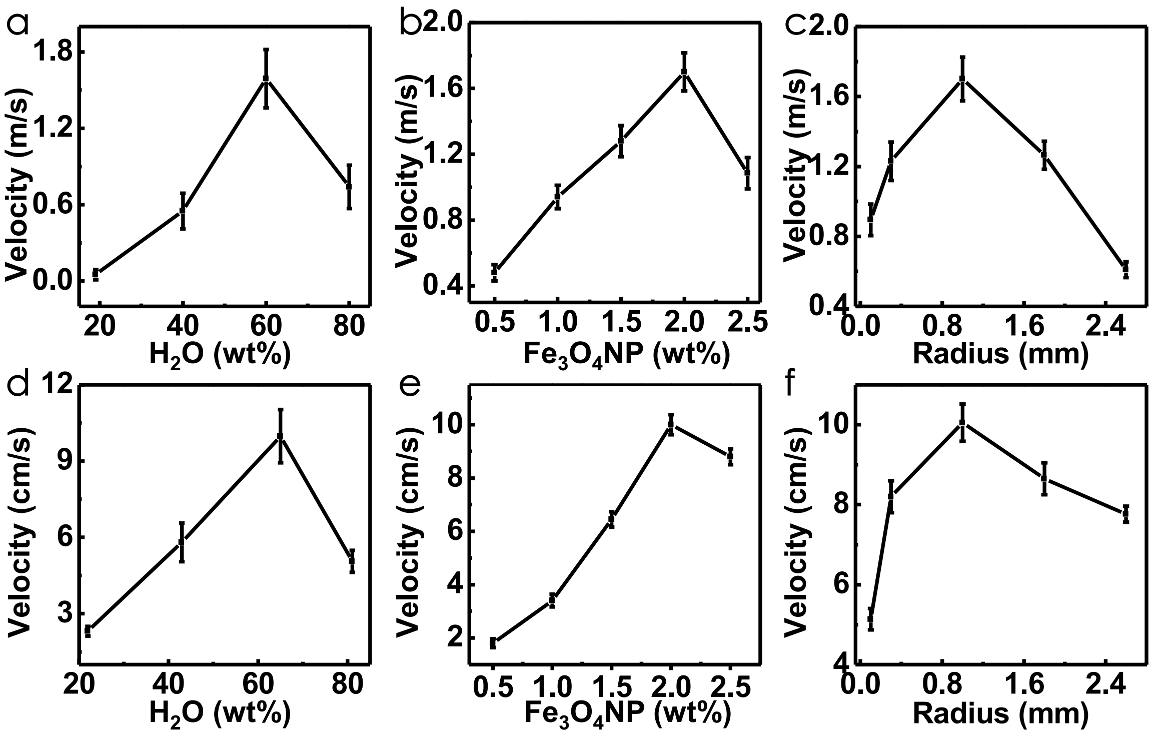

Supplementary Figure 14. The initial velocity of (a-c) jumping (under 2.34 W irradiation) and (d-f) rolling (under 0.67 W irradiation) for hydrogel actuators with different H_2O content, Fe_3O_4NP content and size. Error bars denote the standard deviation.

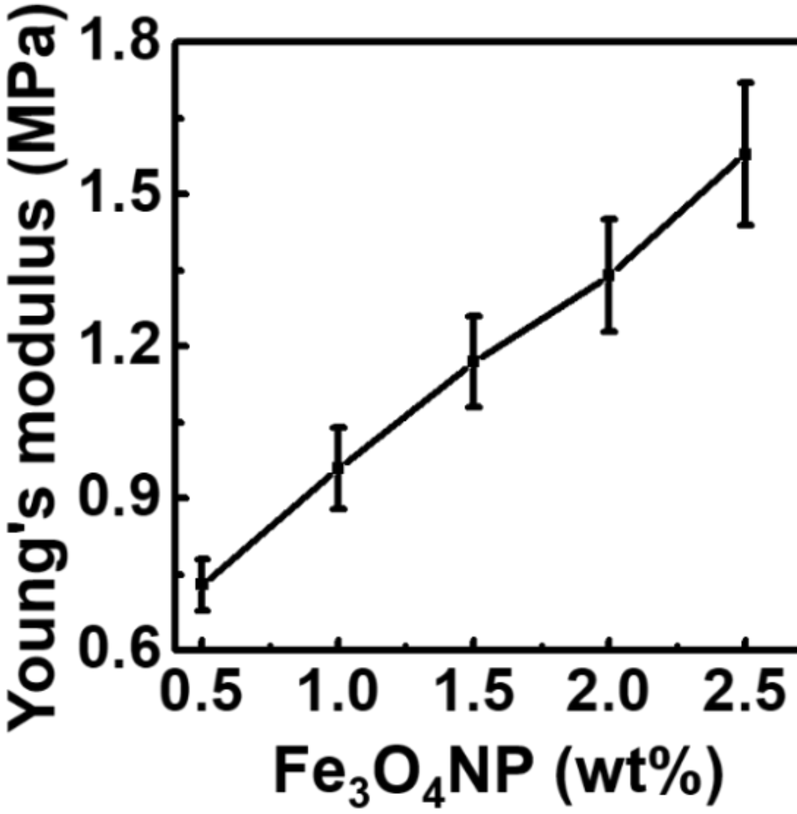

Supplementary Figure 15. Young's modulus of the hydrogel with different IONP content. Error bars denote the standard deviation.

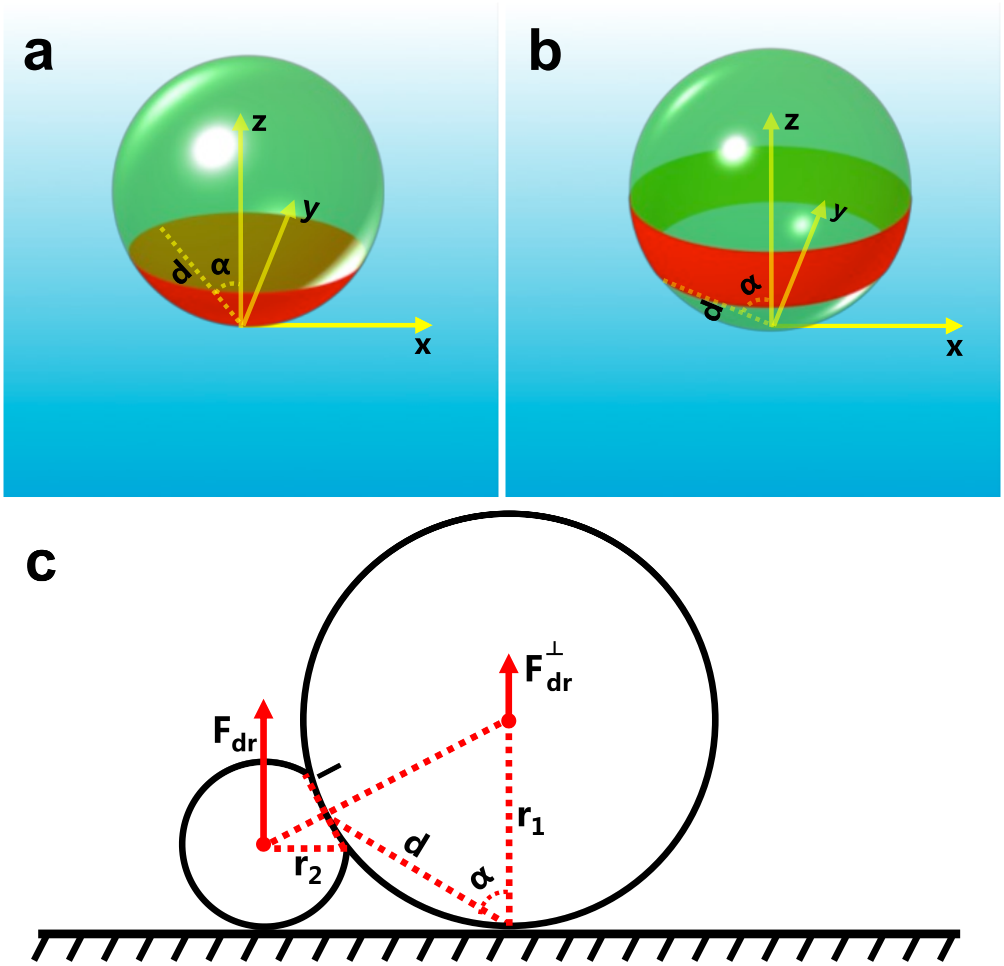

Supplementary Figure 16. Schematics illustrate the light irradiation position (red area) in order to achieve the (a) jumping, (b) rolling motion and (c) the force analysis.

The critical value of α (i.e., the angle between d and the Z-axis) in case of the largest bubble can be calculated by the following equation:

$$cos(180\text{-}2\alpha) = \frac{r_1\text{-}r_2}{r_1+\sqrt{r_2^2\text{-}(\frac{l}{2})^2}}$$

where l is defined as the contact length between the hydrogel actuator and the bubble; r_1 and r_2 are the radius of the hydrogel actuator and the generated bubble, respectively; d is the distance from the bottom to the center of the contact line between the hydrogel actuator and the generated bubble.

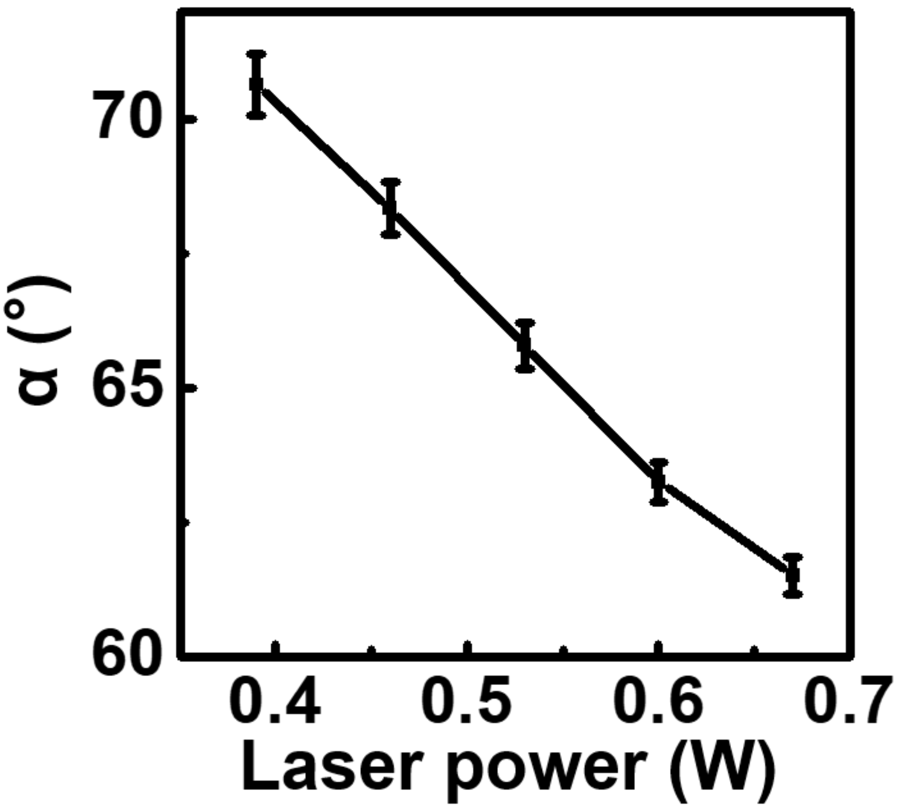

Supplementary Figure 17. The critical value of α depends on the laser power. Error bars denote the standard deviation.

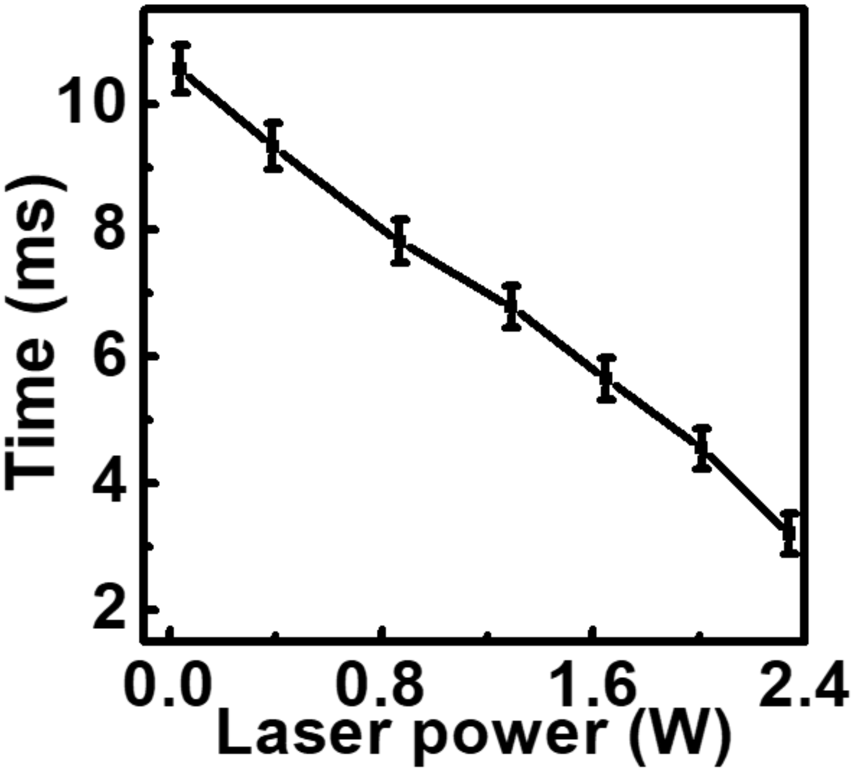

Supplementary Figure 18. The bubble formation time of the hydrogel actuator under light irradiation with different powers. Error bars denote the standard deviation.

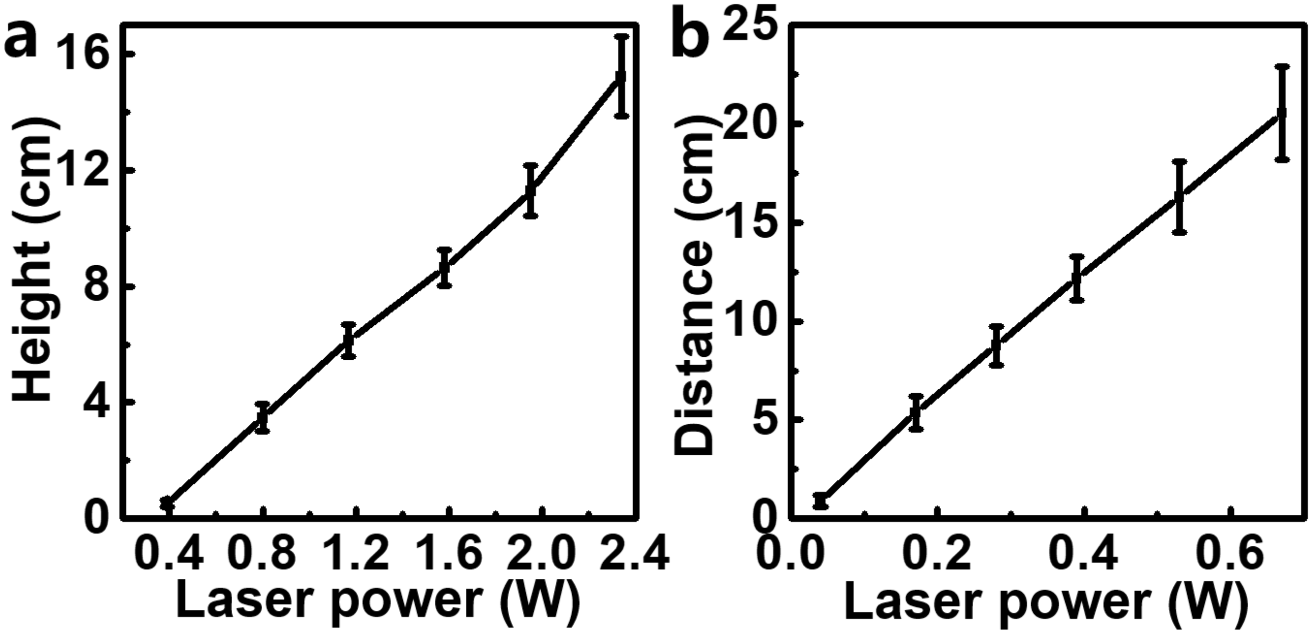

Supplementary Figure 19. (a) The jumping height and (b) rolling distance of hydrogel actuators driven by different laser powers. Error bars denote the standard deviation.

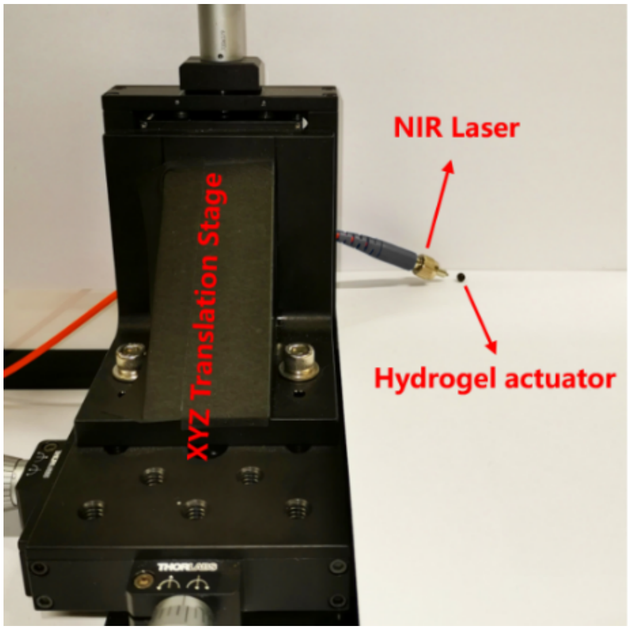

Supplementary Figure 20. CCD camera image showing the experiment setup for the accurate control of the actuator motion by fixing the NIR laser on a XYZ translation stage.

In order to accurately control the trajectory of the actuator, we have fixed the NIR laser on an XYZ translation stage, as shown in Supplementary Fig. 20, and utilized a glass as the flat substrate and made it level with the assistance of a bubble level.

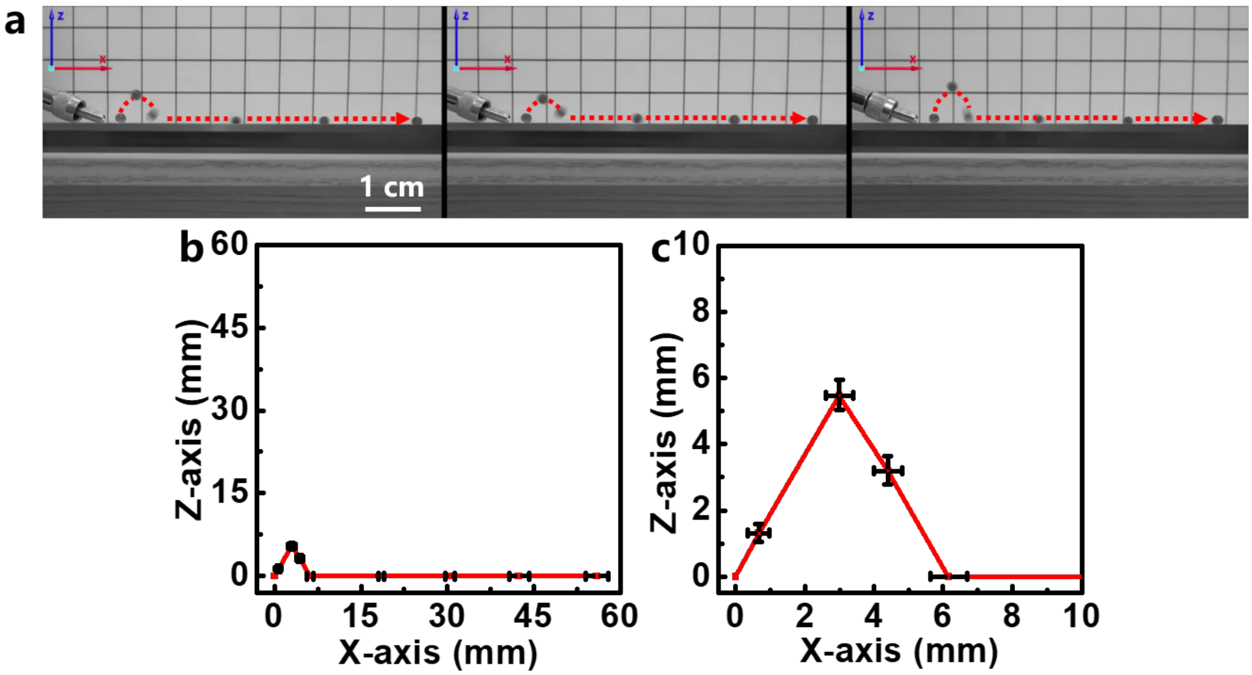

Supplementary Figure 21. (a) Overlaid CCD camera image of the jumping behavior obtained from Supplementary Video 12 and (b) the trajectory analysis from a number of actuators (n = 30) under 0.39 W light irradiation and $\alpha = 70^{\circ}$. (c) The enlarged curve of (b) showing the initial trajectory of the jumping motion. Error bars denote the standard deviation.

As can be seen from the overlaid image (a) which is obtained from Supplementary Video 12 and the moving trajectory analysis from a number of actuators (n = 30) (b-c), the jumping trajectory and destination could be controlled at fixed laser power and α.

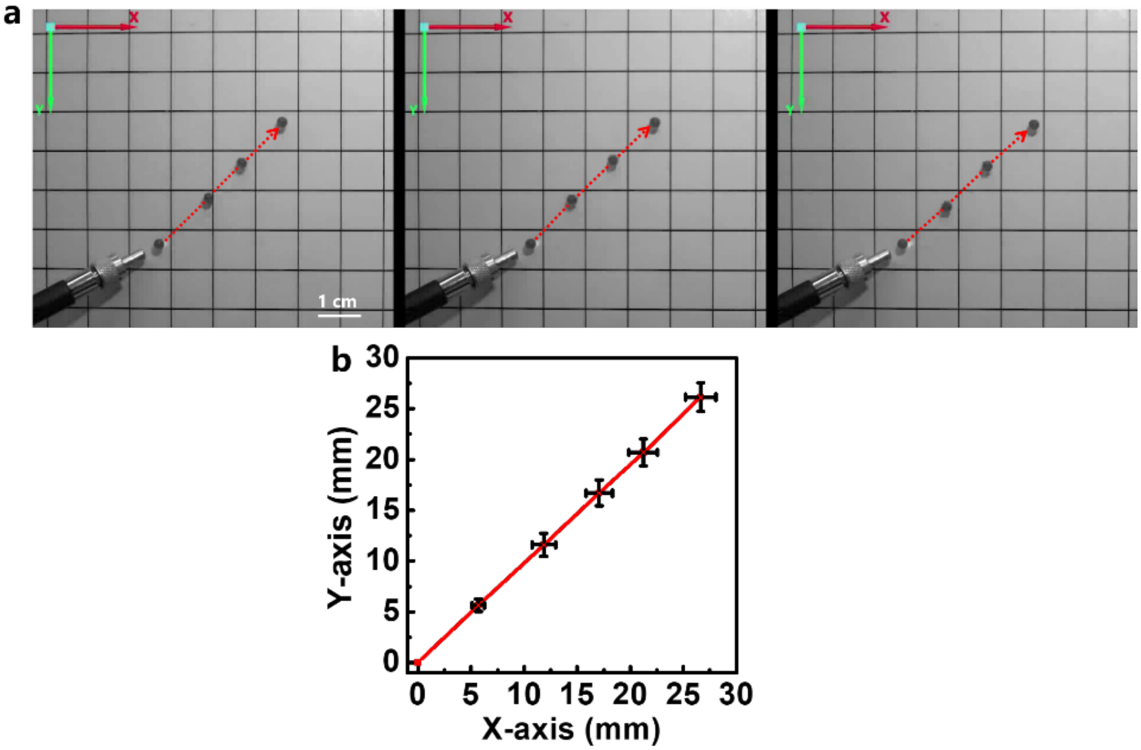

Supplementary Figure 22. (a) Overlaid CCD camera images obtained from Supplementary Video 13 and (b) the trajectory analysis from a number of actuators (n = 30) indicating the rolling behavior of the hydrogel actuator under 0.14 W light irradiation with $\alpha = 60^{\circ}$. Error bars denote the standard deviation.

As can be seen from the overlaid image (a) which is obtained from Supplementary Video 13 and the rolling trajectory analysis from a number of actuators (n = 30) (b), the rolling trajectory and destination can be controlled when the laser power and irradiation position (α) are fixed.

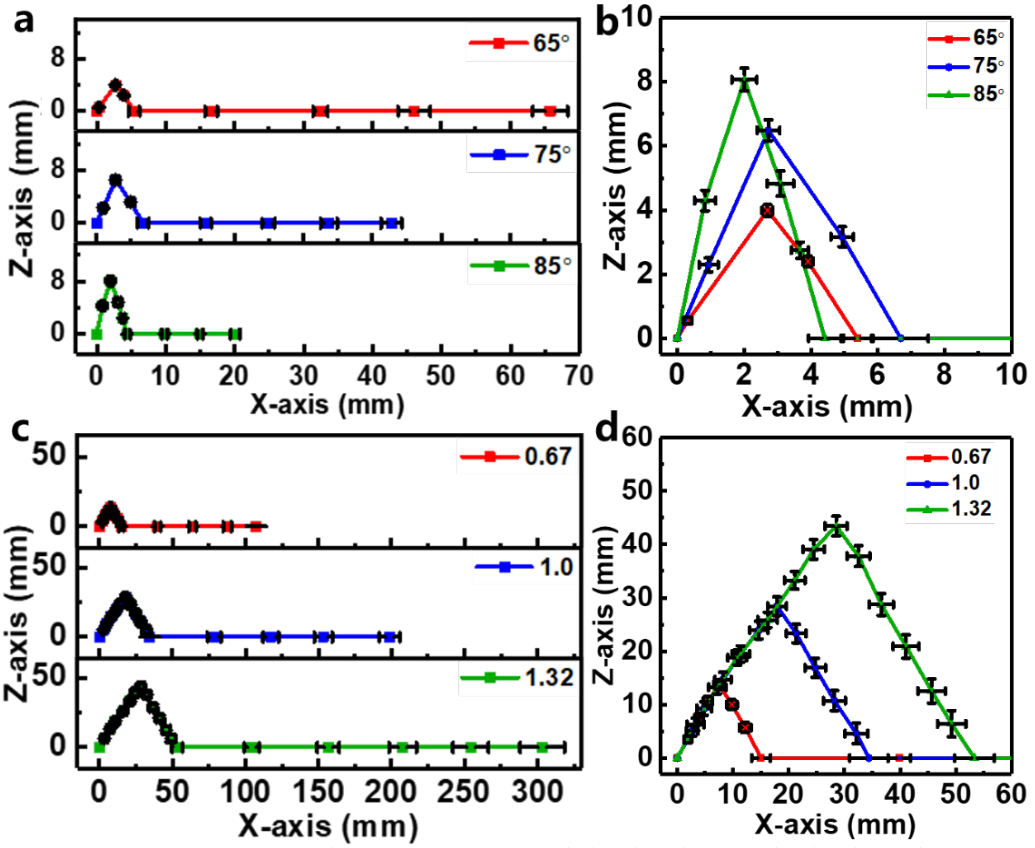

Supplementary Figure 23. (a) Jumping trajectories and destination of the hydrogel actuator under 0.39 W light irradiation when changing α. (b) The enlarged curve of (a) showing the initial trajectory of the jumping motion. (c) Jumping trajectories and destinations of the hydrogel actuator under different laser power (W) when α is fixed at 70°. (d) The enlarged curve of (c) showing the initial trajectory of the jumping motion. Error bars denote the standard deviation.

We have studied the jumping trajectory and destination by changing α. As shown in (a,b), the jumping trajectories and destinations are tunable by adjusting α through varying the irradiation position. Note that the irradiation is fixed at 0.39 W. In addition, the jumping trajectory and destination of the actuator could also be adjusted by changing the laser power (α is fixed at 70°), as can be seen from (c,d). These results (Supplementary Figure 23) indicate that the jumping trajectories and destinations of the hydrogel actuator are controllable by adjusting the laser power and the light irradiation position. Among

others, the irradiation position and the laser power determine the take-off angle and the jumping height, respectively.

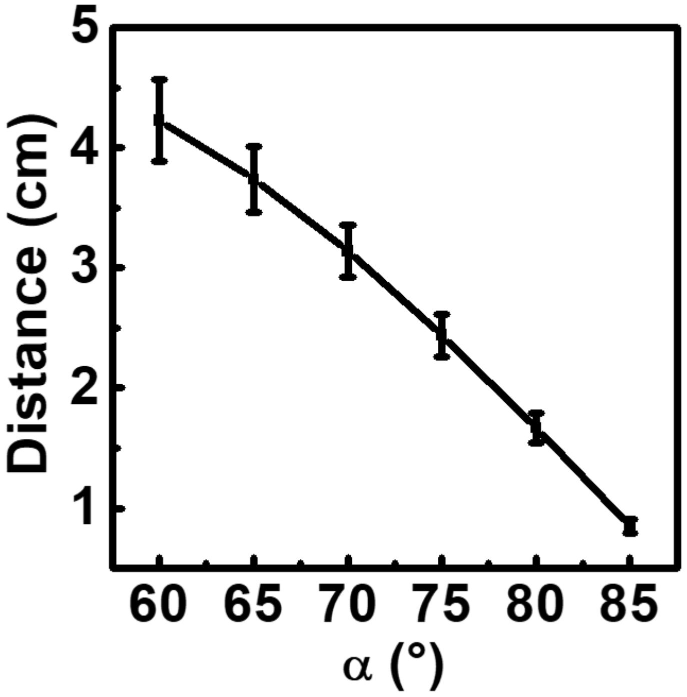

Supplementary Figure 24. Rolling destination of the hydrogel actuator when changing α (laser power is fixed at 0.14 W). Error bars denote the standard deviation.

Furthermore, we have studied the rolling destination by changing α and laser power. As shown in the above figure, the rolling destinations are controllable by adjusting α through varying the irradiation position (at fixed laser power, 0.14 W) or the laser power (at fixed $\alpha = 60^{\circ}$ shown in Supplementary Figure 19b).

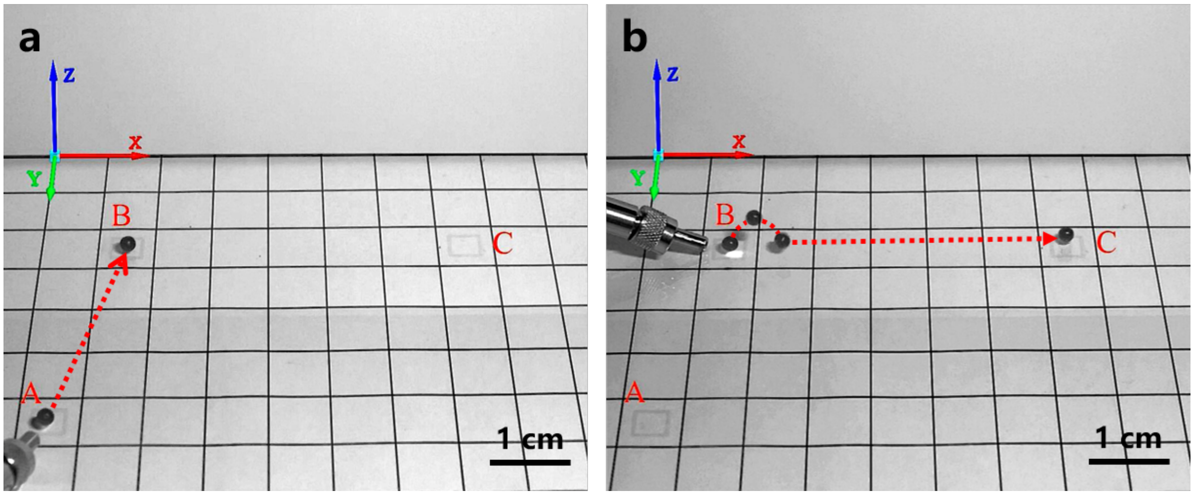

Supplementary Figure 25. Overlaid CCD camera images indicating (a) the rolling of the hydrogel actuator from position A to position B under 0.14 W irradiation and (b) jumping followed by rolling from position B to position C under 0.39 W laser power. These images are captured from Supplementary Video 14.

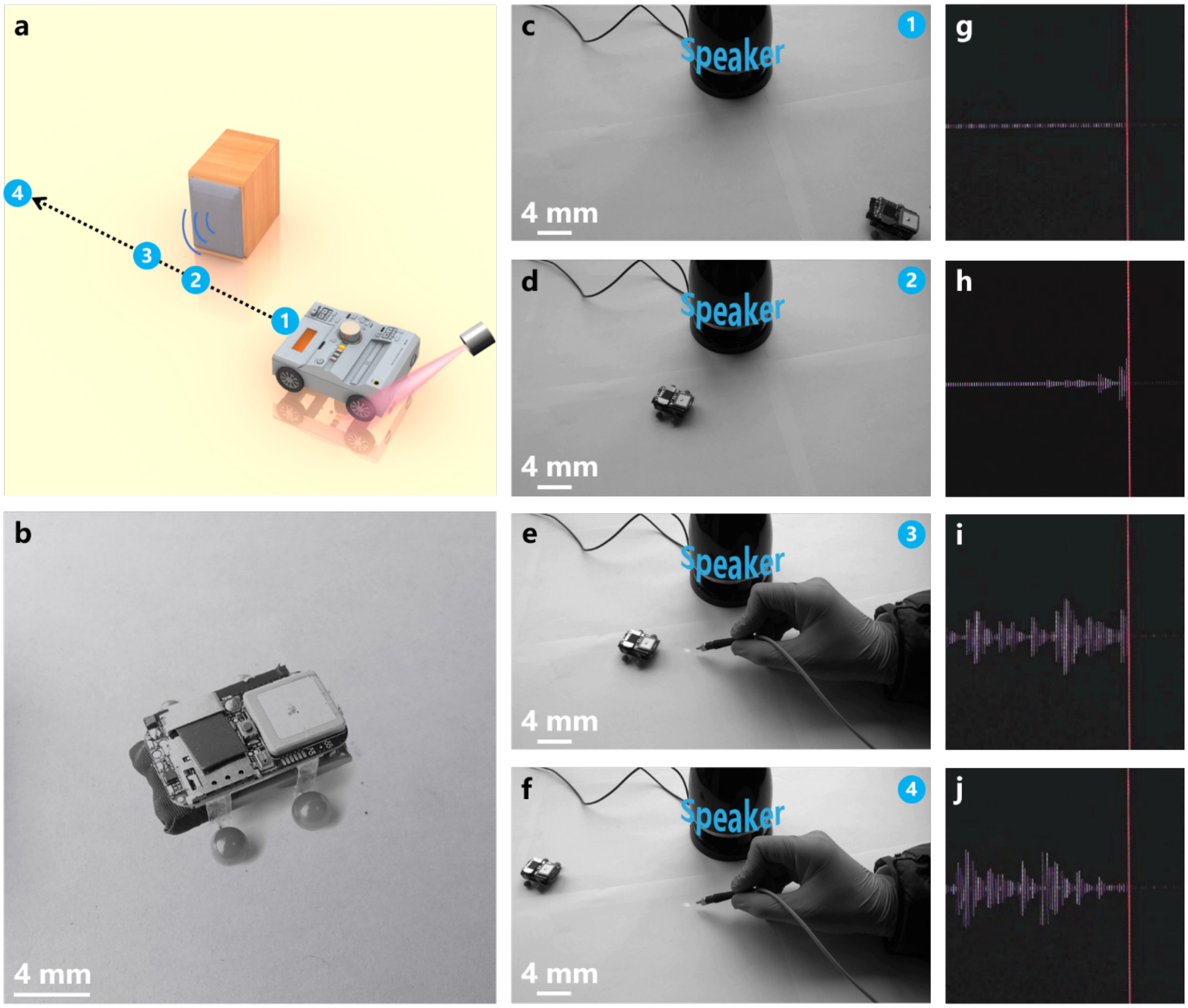

Supplementary Figure 26. The application of the hydrogel actuator as a sound-recording robot. (a) Schematic illustration and (b) the CCD image of the light-driven robot consisting of four wheels made up of hydrogel actuators and a mini detectaphone for sound recording. (c-f) The motion of the robot toward the speaker under 2.34 W irradiation. The number represents the different position of the robot marked in (a). The corresponding recorded sound at different locations (1-4 in (a)) is shown in (g-j), respectively. The corresponding movie is shown in Supplementary Video 19.

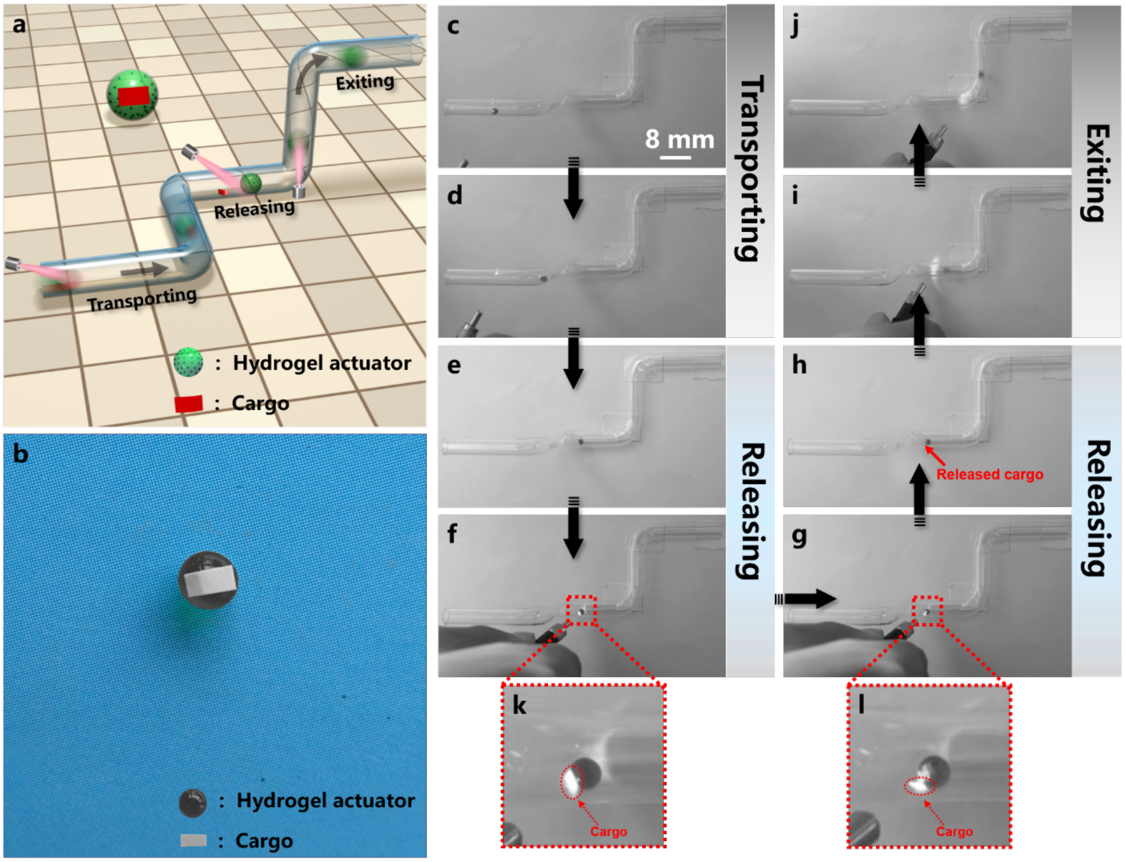

Supplementary Figure 27. (a) Schematic illustration showing the controlled cargo transportation and release based on the light-driven hydrogel actuator. (b) CCD image of the hydrogel actuator with a model cargo consisting of paper attached to part of its surface. The actuator (c-d) transports and (e-h) releases the model cargo inside a glass tunnel before (i-j) its exit (the irradiation is 2.34 W and 0.67 W for jumping and rolling, respectively). (k) and (l) are the enlarged images of (f) and (g), respectively, indicating the cargo releasing process. The corresponding movie is shown in Supplementary Video 20.

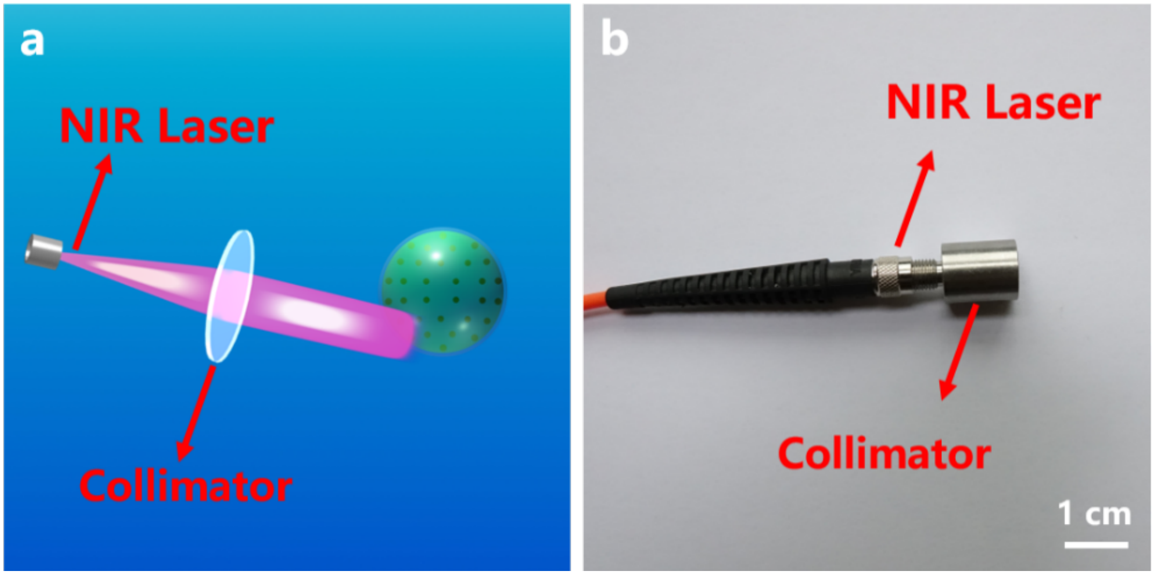

Supplementary Figure 28. (a) Schematic showing the narrowing of the divergent NIR beam through the application of a collimator. (b) CCD camera image of the NIR optical fiber equipped with a collimator at the end.

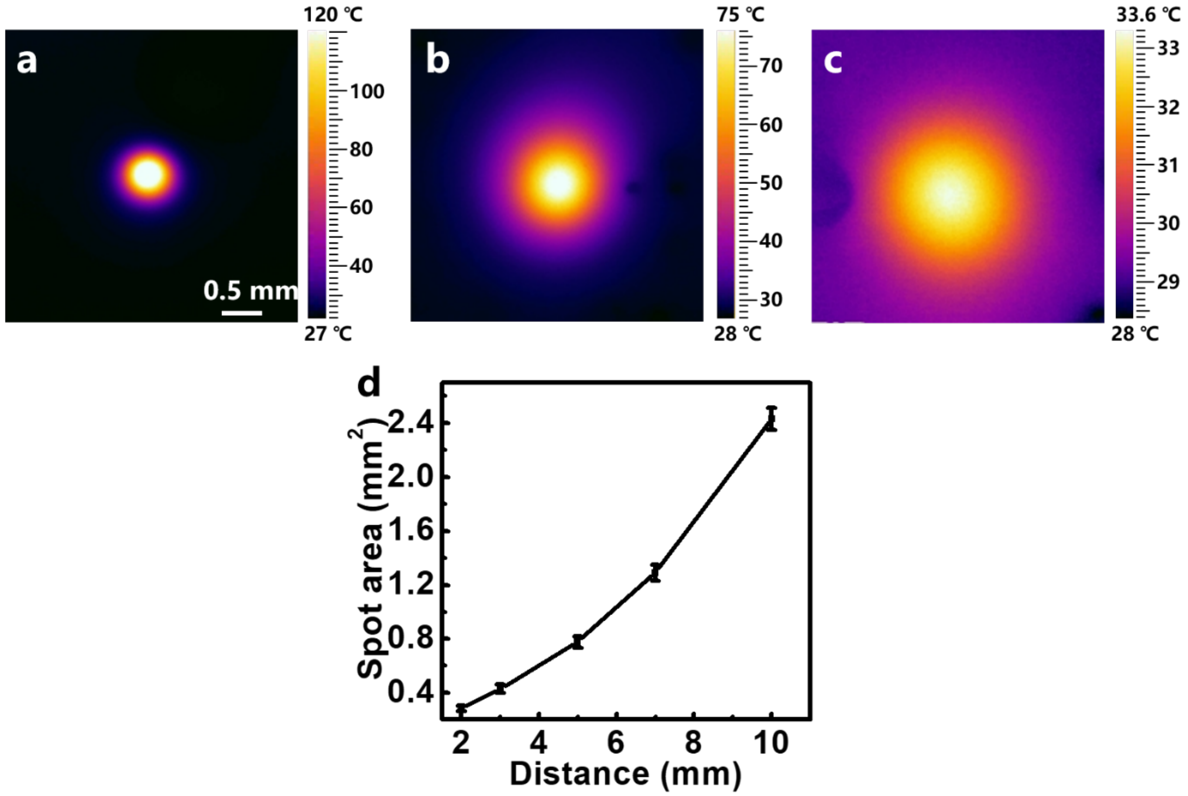

Supplementary Figure 29. Infrared thermal images of the NIR (2.34 W) spot area on the surface of the hydrogel actuator. The distances between the laser and the hydrogel actuator are (a) 2 mm, (b) 7 mm and (c) 10 mm, respectively. (d) The NIR spot area as a function of the distance between the NIR laser and the hydrogel actuator. Error bars denote the standard deviation.

The NIR laser beam is divergent and the spot area is dependent on the distance from the laser to the object. Because of the invisibility of the NIR light, we estimate the laser spot area by measuring the thermal image of the hydrogel actuator under NIR irradiation. As shown in (d), the NIR spot area increases with the increasing distance between the laser and the hydrogel actuator.

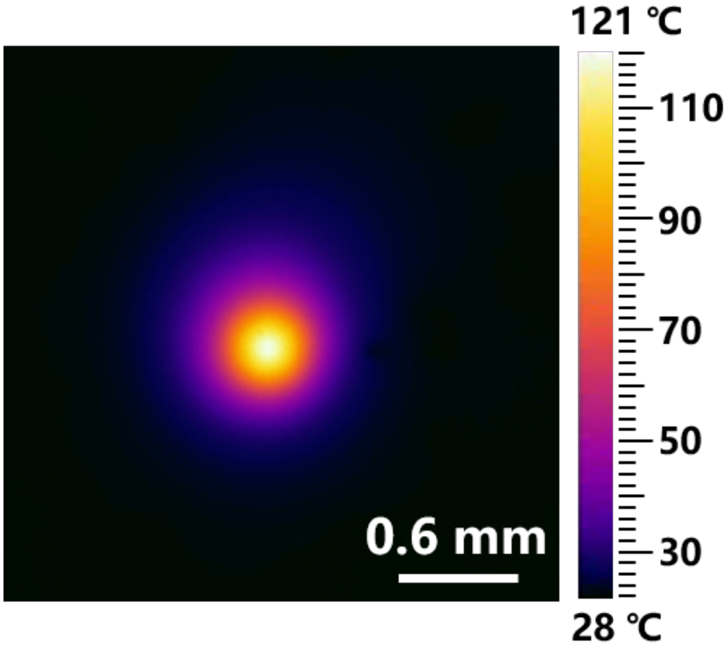

Supplementary Figure 30. Infrared thermal images of NIR light spot on the hydrogel actuator when the distance between the NIR optical fiber with a collimator end and the actuator is 35 mm under 2.34 W irradiation.

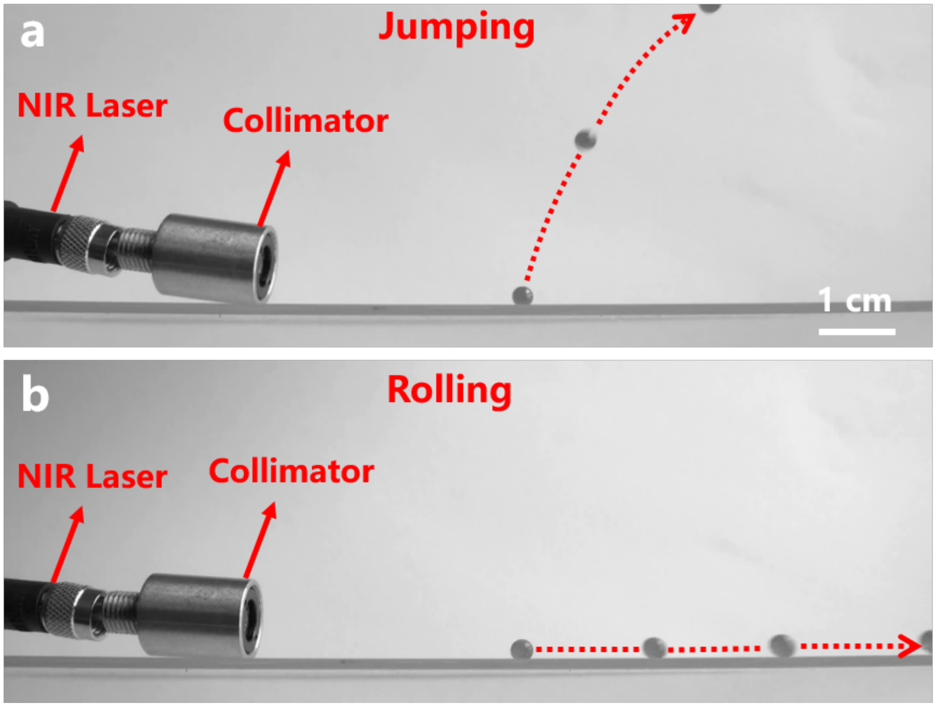

Supplementary Figure 31. (a) Jumping (2.34 W irradiation) and (b) rolling (0.67 W irradiation) behavior of the hydrogel actuator actuated by the NIR light (equipped with a collimator) which is placed 35 mm away from the actuator. (a) and (b) are overlaid images captured from Supplementary Video 21 and Video 22, respectively.

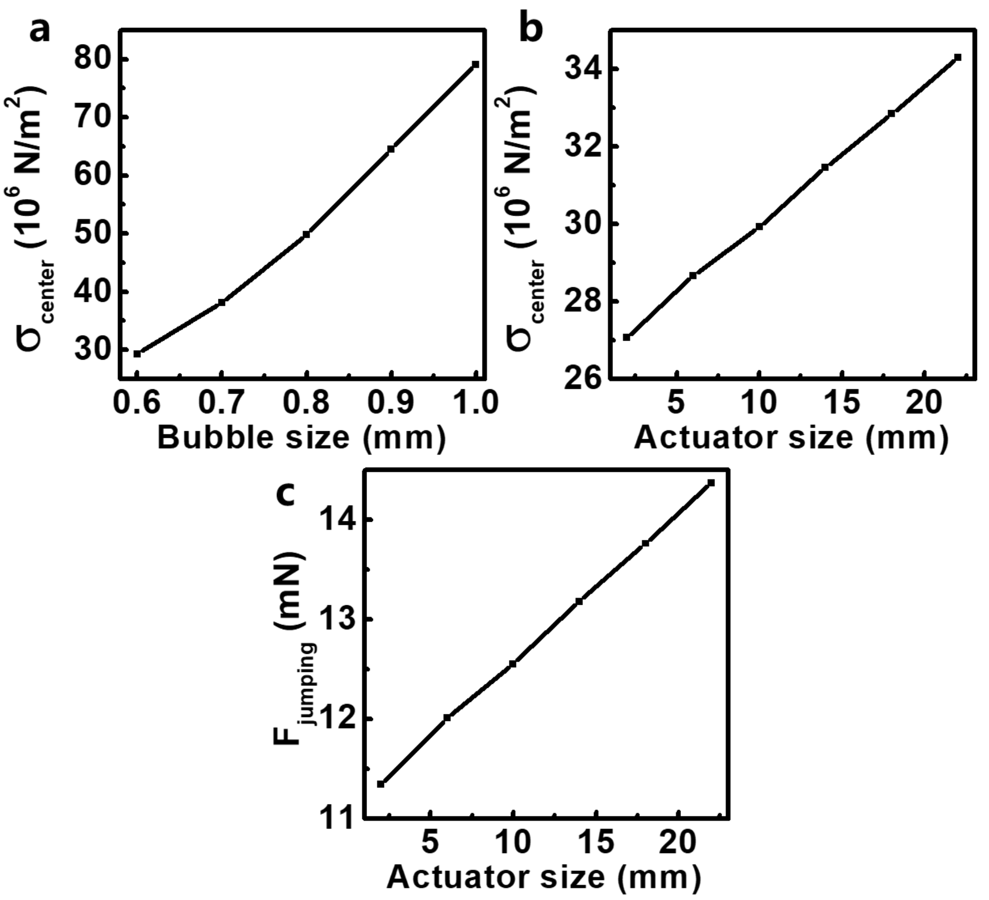

Supplementary Figure 32. (a) σ_{center} as a function of the bubble size when the actuator size is fixed at 2 mm. (b) σ_{center} and (c) $F_{jumping}$ as a function of the actuator size when the bubble size is fixed at 0.6 mm.

The relation between contact pressure and bubble size is obtained through the simulation method. In order to successfully perform the simulation, different parameters, such as the bubble size, the actuator size, etc., need to be inputted ahead of time. In addition, as shown in the Supplementary Figure 9, the contact pressure is different inside the contact area. Thus, we have studied the contact pressure at the center of the contact area (σ_{center}) as a function of the bubble size (at the fixed actuator size, 2 mm) and the actuator size (when the bubble size is fixed at 0.6 mm) through the simulation method. As can be seen

from (a-b), σ_{center} increases with the increasing bubble size and actuator size. The relation between $F_{jumping}$ and the actuator size could be obtained based on equation 2, as shown in (c).

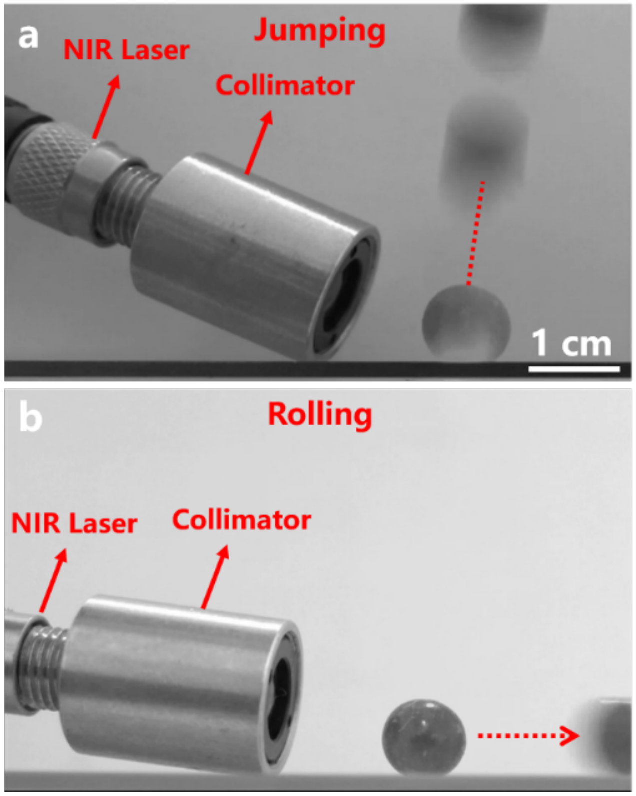

Supplementary Figure 33. (a) Jumping (2.34 W irradiation) and (b) rolling (0.67 W irradiation) behavior of the big (8 mm diameter) hydrogel actuator actuated by the NIR light (equipped with a collimator). (a) and (b) are overlaid images captured from Supplementary Video 23 and Video 24, respectively.

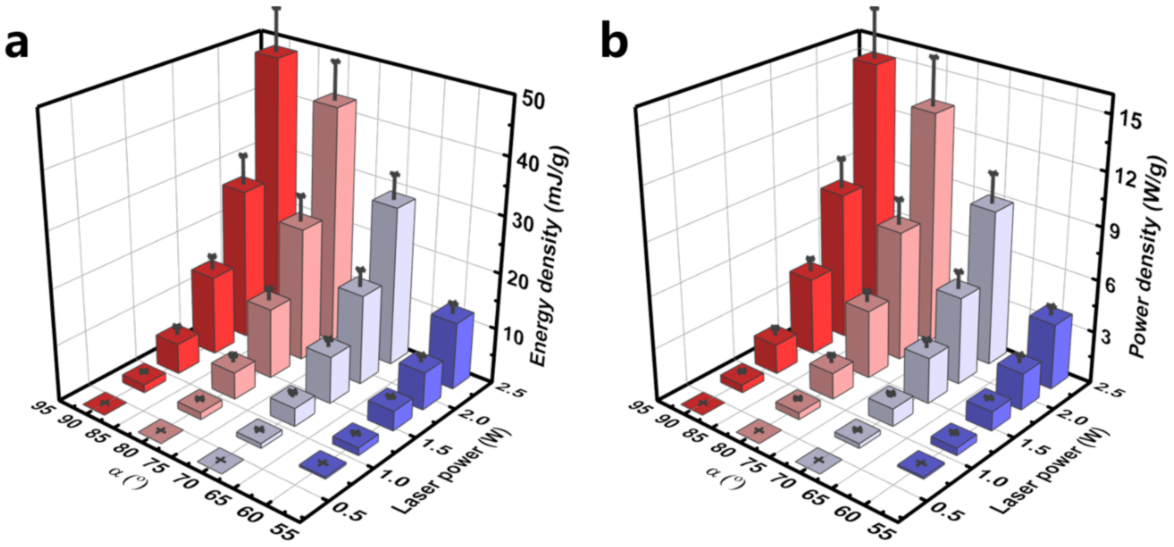

Supplementary Figure 34. The estimated (a) energy densities and (b) power density of jumping at different laser powers and α. Error bars denote the standard deviation.

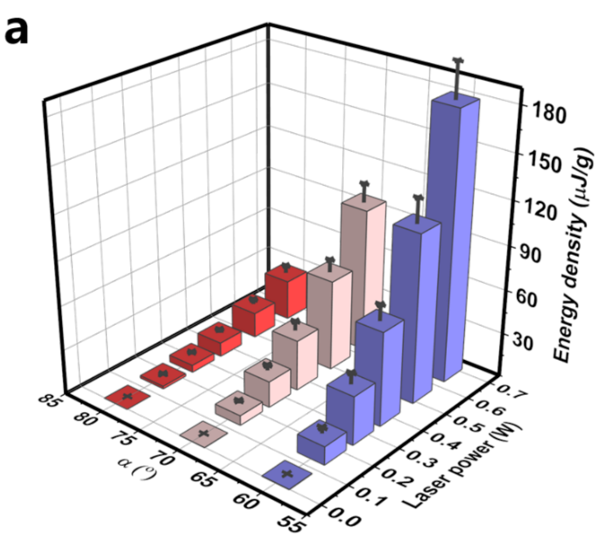

Supplementary Figure 35. The estimated (a) energy densities and (b) power densities of rolling at different laser powers and α. Error bars denote the standard deviation.

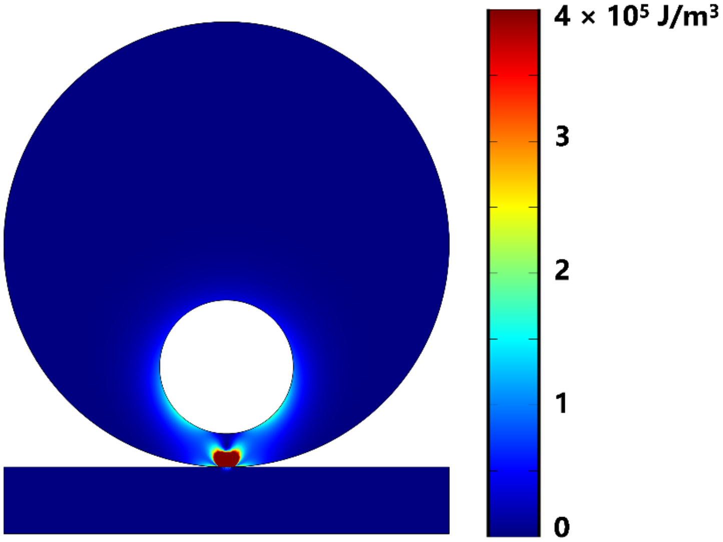

Supplementary Figure 36. The strain energy density of the shape deformation of the hydrogel actuator obtained by using the numerical simulation.
