# Supplementary material for: In-air fast response and high speed jumping and rolling of a light-driven hydrogel actuator
Source: Nat Commun. 2020 Aug 10;11:3988. doi: 10.1038/s41467-020-17775-4 (PMC7417580; doi:10.1038/s41467-020-17775-4)
Supplement: Supplementary file 3 — Description of Additional Supplementary Files [file 41467_2020_17775_MOESM3_ESM.pdf]

Description of Additional Supplementary Files

File Name: Supplementary Movie 1
Description: The jumping behavior of the hydrogel actuator when illuminating its bottom part using 2.34 W laser.

File Name: Supplementary Movie 2
Description: The rolling behavior of the hydrogel actuator by irradiating its side surface (under 0.67 W irradiation).

File Name: Supplementary Movie 3
Description: The take-off process of the hydrogel actuator recorded by the highspeed camera (under 2.34 W laser irradiation).

File Name: Supplementary Movie 4
Description: The surfacing and diving of the hydrogel actuator upon 2.34 W NIR actuation.

File Name: Supplementary Movie 5
Description: The bubble production and restoration of a hydrogel actuator fixed at a needle end under 2.34 W NIR irradiation.

File Name: Supplementary Movie 6
Description: The take-off process of the hydrogel actuator without PBR surface coating recorded by the high-speed camera (under 2.34 W laser irradiation)

File Name: Supplementary Movie 7
Description: Video captured by the high-speed camera showing the shape deformation before rolling (under 0.67 W laser irradiation).

File Name: Supplementary Movie 8
Description: The hydrogel actuator jumps to the left by irradiating its bottom right part (under 2.34 W NIR irradiation).

File Name: Supplementary Movie 9
Description: The hydrogel actuator jumps to the right side by illuminating its bottom left position (under 2.34 W NIR irradiation).

File Name: Supplementary Movie 10
Description: The hydrogel actuator rolls to the left by irradiating its right side surface with 0.67 W laser.

File Name: Supplementary Movie 11
Description: The hydrogel actuator rolls to the right side, which is realized by irradiating its left side surface with 0.67 W laser.

File Name: Supplementary Movie 12
Description: The controllability of the jumping trajectory and destination of the hydrogel actuator under 0.39 W light irradiation with $\alpha = 70°$.

File Name: Supplementary Movie 13
Description: The controllability of the rolling destination of the hydrogel actuator under 0.14 W light irradiation with $\alpha = 60°$

File Name: Supplementary Movie 14
Description: Movement of the hydrogel actuator from position A to the designated position B through rolling (under 0.14 W irradiation) and to the designated position C through jumping and rolling motion (under 0.39 W irradiation).

File Name: Supplementary Movie 15
Description: The hydrogel actuator jumps across a barrier under 2.34 W NIR actuation.

File Name: Supplementary Movie 16
Description: The hydrogel actuator rolls over an incline under 0.67 W laser actuation.

File Name: Supplementary Movie 17
Description: The hydrogel actuator approaches (under 0.1 W irradiation) and passes through a narrow slit (under 0.19 W irradiation).

File Name: Supplementary Movie 18
Description: The hydrogel moves through a pellucid Z-pipe based on the combined rolling (under 0.67 W laser actuation) and jumping (under 2.34 W laser actuation) motion.

File Name: Supplementary Movie 19
Description: The hydrogel actuator functions as a sound-recording robot under 2.34 W NIR actuation.

File Name: Supplementary Movie 20
Description: Controlled cargo transportation and release based on the hydrogel actuator (2.34 W and 0.67 W irradiation for jumping and rolling, respectively).

File Name: Supplementary Movie 21
Description: . Jumping behavior of the hydrogel actuator actuated from a longer operation distance (2.34 W irradiation).

File Name: Supplementary Movie 22
Description: Rolling behavior of the hydrogel actuator actuated from a longer operation distance (0.67 W irradiation).

File Name: Supplementary Movie 23
Description: Jumping behavior of a bigger sized hydrogel actuator under 2.34 W NIR actuation.

File Name: Supplementary Movie 24
Description: . Rolling behavior of a bigger sized hydrogel actuator driven by 0.67 W laser.
